# Measuring disease similarity and predicting disease-related ncRNAs by a novel method

**DOI:** 10.1186/s12920-017-0315-9

**Published:** 2017-12-28

**Authors:** Yang Hu, Meng Zhou, Hongbo Shi, Hong Ju, Qinghua Jiang, Liang Cheng

**Affiliations:** 1 School of Life Science and Technology, Harbin Institute of Technology, Harbin, 150001 People’s Republic of China; 2 College of Bioinformatics Science and Technology, Harbin Medical University, Harbin, 150001 China; 3 Department of information engineering, Heilongjiang biological science and technology Career Academy, Harbin, 150001 China

**Keywords:** Information flow, Disease similarity, Gene functional network, lncRNA similarity network

## Abstract

**Background:**

Similar diseases are always caused by similar molecular origins, such as diasease-related protein-coding genes (PCGs). And the molecular associations reflect their similarity. Therefore, current methods for calculating disease similarity often utilized functional interactions of PCGs. Besides, the existing methods have neglected a fact that genes could also be associated in the gene functional network (GFN) based on intermediate nodes.

**Methods:**

Here we presented a novel method, InfDisSim, to deduce the similarity of diseases. InfDisSim utilized the whole network based on random walk with damping to model the information flow. A benchmark set of similar disease pairs was employed to evaluate the performance of InfDisSim.

**Results:**

The region beneath the receiver operating characteristic curve (AUC) was calculated to assess the performance. As a result, InfDisSim reaches a high AUC (0.9786) which indicates a very good performance. Furthermore, after calculating the disease similarity by the InfDisSim, we reconfirmed that similar diseases tend to have common therapeutic drugs (Pearson correlation γ^2^ = 0.1315, *p* = 2.2e-16). Finally, the disease similarity computed by infDisSim was employed to construct a miRNA similarity network (MSN) and lncRNA similarity network (LSN), which were further exploited to predict potential associations of lncRNA-disease pairs and miRNA-disease pairs, respectively. High AUC (0.9893, 0.9007) based on leave-one-out cross validation shows that the LSN and MSN is very appropriate for predicting novel disease-related lncRNAs and miRNAs, respectively.

**Conclusions:**

The high AUC based on benchmark data indicates the method performs well. The method is valuable in the prediction of disease-related lncRNAs and miRNAs.

**Electronic supplementary material:**

The online version of this article (doi: 10.1186/s12920-017-0315-9) contains supplementary material, which is available to authorized users.

## Background

One way to indicate the associations between pair-wise diseases in quantitatively is their similarity. In comparison with the associations, disease similarity can indicate the relationships between diseases of multiple categories more clearly and easily, for instance, cancers [1]. In the previous studies, disease similarity was exploited to compute similarities between protein-coding RNA genes (PCGs), which can help to disclose the complex pathogenesis of diseases [1]. Moreover, disease similarity was also employed to calculate similarities between microRNA genes (miRNAs) [2, 3], and long non-coding RNA genes (lncRNAs) [4–8], respectively, which could be applied for constructing functional network of non-coding RNA genes (ncRNAs). Recently, similarity between diseases was even utilized to predict potential therapeutic drugs for diseases [9–12].

Semantic associations and disease gene associations are often considered to be quantitative for evaluating disease similarity. Semantic associations between diseases were documented in the ontology around disease terms. The most widely used ontology for calculating disease similarity is Disease Ontology (DO) [13], which is the first ontology to be established around disease terms. DO defines a type of semantic association named ‘IS_A’ relationship, which reflects set inclusion relationships between disease terms [14]. Disease terms of DO could build a directed acyclic graph (DAG) based on the ‘IS_A’ relationship. Disease-related genes were distributed in different sources, such as Comparative Toxicogenomics Database (CTD) [15], Online Mendelian Inheritance in Man (OMIM) [16], Gene Reference into Functions (GeneRIFs) [17], Genetic Association Database (GAD) [18], and so on.

Three widely used methods for computing the similarity of terms of ontology were presented by Resnik [19], Lin [20], and Wang et al. [21] repectively. All of these three methods were utilized for computing disease similarity by DOSim [1]. Resnik presented Information content (IC) of terms of ontology [19], and in this method, IC of the most informative common ancestor (MICA) of pair-wise diseases was served as the similarity of them. Due to the IC of the pair-wise terms and the IC of the MICA could contribute to the similarity of them, Lin [20] improved Resnik’s method. By the contrast of Resnik’s and Lin’s method, Wang et al. [21] computed the similarity between terms fully based on semantic associations of terms in ontology.

In recent years, three methods for calculating similarity of terms of DO were presented. Disease-related genes have been the focus of all these methods. In another word, the similarity of two diseases was converted to the similarity of the two gene sets of diseases. Mathur and Dinakarpandian first presented to utilize the figure of overlapping genes to calculate disease similarity [22]. Even though two gene sets have no shared genes, these two sets could also be connected by their presence during the same or similar biological process. Therefore, Mathur and Dinakarpandian designed a process-similarity based (PSB) method to compute disease similarity based on biological process terms of Gene Ontology [23, 24]. Besides biological process, co-expression [25] and protein-protein interaction [26] could also be employed to similarity of disease-related gene sets [27, 28]. Hence, Cheng et al. combined semantic association and the comprehensive gene functional network to compute disease similarity (SemFunSim) [11], which performs very well.

Improved knowledge has suggested that semantic associations and disease gene associations are two types of significant associations, which were widely exploited to measure disease similarity. Recent studies focused on incorporating disease gene associations from different views. Eventually, comprehensive gene functional network (GFN) was incorporated in SemFunSim method [11], in which functional interactions of pair-wise genes were considered. Obviously, it is straightforward to consider that whether the entire network could be completely utilized to measure disease similarity. For this purpose, we designed a novel method, called *InfDisSim*, to figure out disease similarity by modeling the information flow in the comprehensive GFN in this study.

## Methods

### Date source

#### Disease ontology

Disease terms and semantic associations were originated from DO [13] (Table 1), which is manually curated for diseases names. As for now, it includes 7124 ‘IS_A’ relationships between 6920 terms.

**Table 1:** Data sources

Data source	Web site (Date of download)
DO	http://disease-ontology.org/ (Jun 2016)
CTD	http://ctdbase.org/ (Jun 2016)
GeneRIF	http://www.ncbi.nlm.nih.gov/gene/about-generif (Jun 2016)
GAD	https://geneticassociationdb.nih.gov/ (Jun 2016)
OMIM	http://www.omim.org/ (Jun 2016)
HumanNet	http://www.functionalnet.org/humannet/download.html (Jun 2016)
LncRNADisease	http://www.cuilab.cn/lncrnadisease (Jun 2016)

#### Disease gene association network

Disease-related genes are derived from the latest version of diversed open source sources involving CTD [15], GAD [18], GeneRIFs [17], and OMIM [16]. Disease terms in these databases were distributed to DO according to SIDD [29]. After integrating all of these four widely used sources, 130,144 associations between 3178 disease terms and 11,717 genes were obtained as disease gene association network (Additional file 1).

#### Comprehensive gene functional network

Comprehensive GFN was estimated from HumanNet [30], which is built around *Homo sapiens*. Multiple interactions spanning human mRNA co-expression, protein-protein interaction, protein complex, and comparative genomics data sets, combining with alike lines of evidence from orthologs in yeast, fly and worm are comprehensively analyzed for the network utilizing a probabilistic method. Currently, it contains 476,399 interactions among 16,243 genes [30].

#### Disease-related drugs

Disease-related drugs were derived from robust, publicly accessible databases CTD [15], which elucidates the process that chemicals affect human health. Disease terms in CTD were distributed to DO according to SIDD [29]. As a result, 16,639 associations between 1093 diseases and 3887 drugs were obtained.

#### Disease-related lncRNAs

Human lncRNA-disease associations [31–36] were incorporated into the lncRNA similarity network (LSN), which was constructed based on disease similarity, to predict potential relationships between diseases and lncRNAs. These associations were derived from a manually curated database LncRNADisease [37], which provided experimentally supported disease-lncRNA associations. After removing disease terminologies not in DO and deploying of duplicate associations, 602 associations between 167 diseases and 338 lncRNAs were obtained (Additional file 2).

#### Disease-related miRNAs

Disease-related human miRNAs were extracted from the Human microRNA Disease Database (HMDD) v2.0 [3]. After manually mapping disease terms of HMDD to DO, we got 5710 associations between 556 miRNAs and 265 diseases (Additional file 3).

### Method for calculating disease similarity

In this study, we designed a novel method to compute disease similarity by modelling the information flow in the comprehensive GFN. In the previous study, a tool called ITM Probe [38] was created for analyzing information flow in the network based on random walk with damping. Currently, three models involving absorbing, emitting, and channel were employed in ITM Probe. According to these three models [39], the initial nodes which are the starting points of the random walk and the sink nodes which are the ending points of the random walk are regarded as boundary nodes, and the rest of the nodes in the network are regarded as transient nodes. Channel model [39] was designed for directed information flow, which extends absorbing model that specify the source of the information flow and emitting model that distributes end of information flow.

**Fig. 1:**
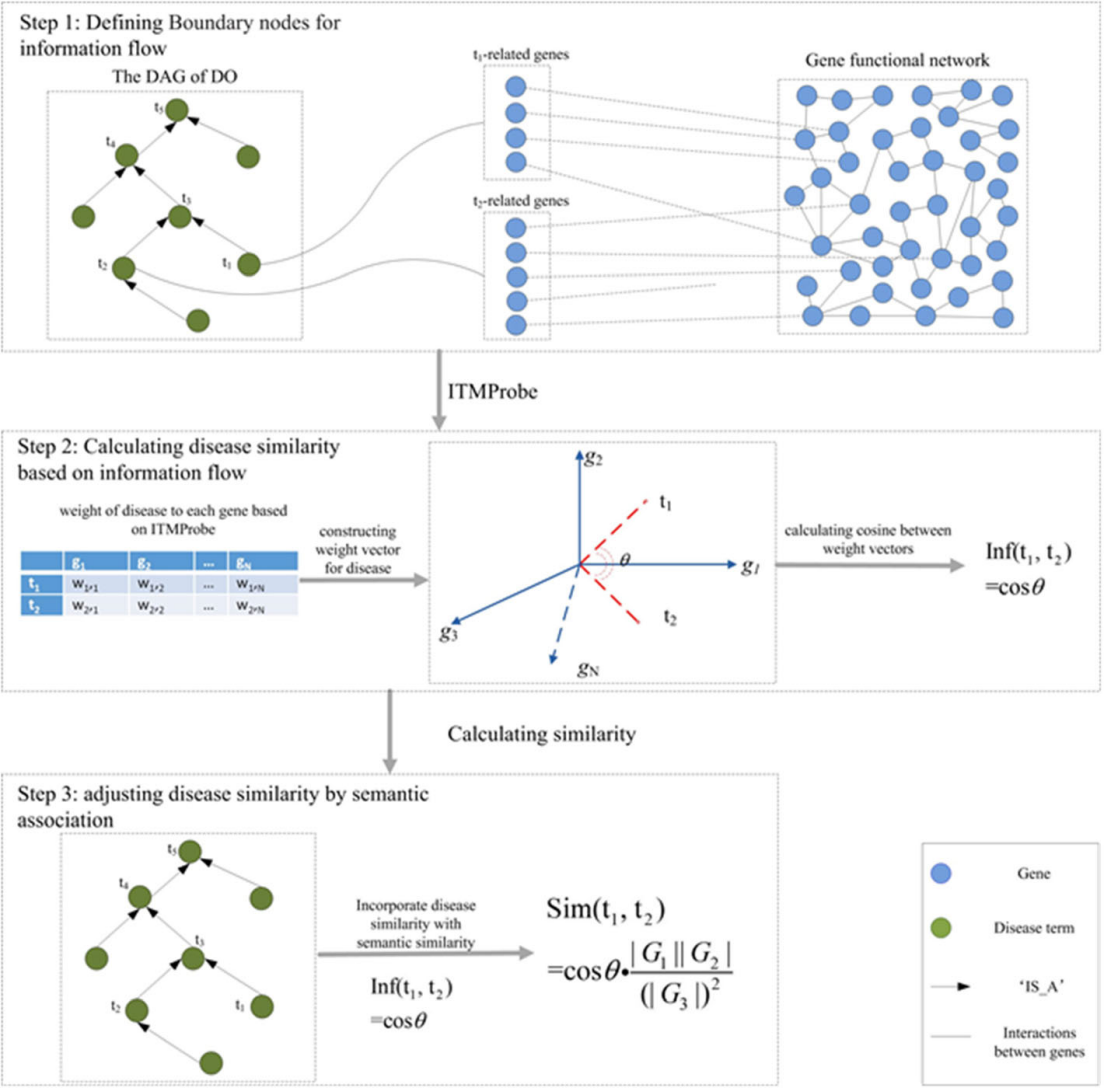
Workflow of InfDisSim to demonstrate the basic ideas of measuring disease similarity

Here, channel model was employed to the network involving disease gene association network and the comprehensive GFN. In this network, disease terms couldn’t be directly linked to each other, however, they could be associated based on their related genes. According to Fig. 1, diseases in the network were considered as boundary nodes, and all the genes were considered as transient nodes. To distribute a weight to each transient nodes for disease, a given disease was considered as both the source node and the sink node in the information flow, and damping factor was distributed as 0.85 based on previous study [39]. Assuming *N* genes exist in the integrative network. Each disease can be represented as *N*-dimension vector based on the ITM Probe. For a give disease *t*_*1*_, the weight vector can be described as:

1 $$ {WV}_{t_1}=\left\{{w}_{1,1},{w}_{1,2},\dots, {w}_{1,i},\dots, {w}_{1,N}\right\}, $$

where ${\mathrm{WV}}_{t_1}$ indicates a weight vector of *t*_*1*_, and *w*_1, *i*_ indicates the weight score of *t*_*1*_ on the *i*th dimension. Then, disease similarity based on the information flow could be defined as the cosine of their vectors as following:

2 $$ \mathrm{Inf}\left({t}_1,{t}_2\right)=\frac{\sum \limits_{i=1}^N{w}_{1,i}\cdot {w}_{2,i}}{\sqrt{\sum \limits_{i=1}^N{w_{1,i}}^2}\sqrt{\sum \limits_{j=1}^N{w_{2,j}}^2}}. $$

Because disease similarity could be reflected by semantic associations and the disease gene associations, the disease similarity is defined as following:

3 $$ \mathrm{InfDisSim}\left({t}_1,{t}_2\right)=\mathrm{Inf}\left({t}_1,{t}_2\right)\frac{\mid {G}_1\Big\Vert {G}_2\mid }{{\left(|{G}_{MICA}|\right)}^2}, $$

where *G*_1_, *G*_2_ indicates gene set of *t*_*1*_ and *t*_*2*_, respectively. *G*_*MICA*_ is the gene set of *t*_*3*_, which is the most informative common ancestor of *t*_*1*_ and *t*_*2*_. And ∣.∣ represents the number of terms in the specified set.

According to Lin’s research, the definition of similarity between pair of terms of DO is as following:

4 $$ Sim\left({t}_1,{t}_2\right)=\frac{2\times IC\left({t}_{MICA}\right)}{IC\left({t}_1\right)+ IC\left({t}_2\right)}, $$

or

5 $$ Sim\left({t}_1,{t}_2\right)=\frac{\log \frac{{\left|{G}_{root}\right|}^2}{{\left|{G}_{MICA}\right|}^2}}{\log \frac{{\left|{G}_{root}\right|}^2}{\mid {G}_1\mid \cdot \mid {G}_2\mid }}, $$

where *G*_*root*_ represents gene sets of the root node of the DAG of DO. According to the eq. 5, the semantic similarity between *t*_1_ and *t*_2_ is proportional to ∣*G*_1_∣ and ∣*G*_2_∣, and is inversely proportional to ∣*G*_*MICA*_∣. Therefore, the proportional relation of Eq. 3 is consistent with the proportional relation of Lin’s method.

**Fig. 2:**
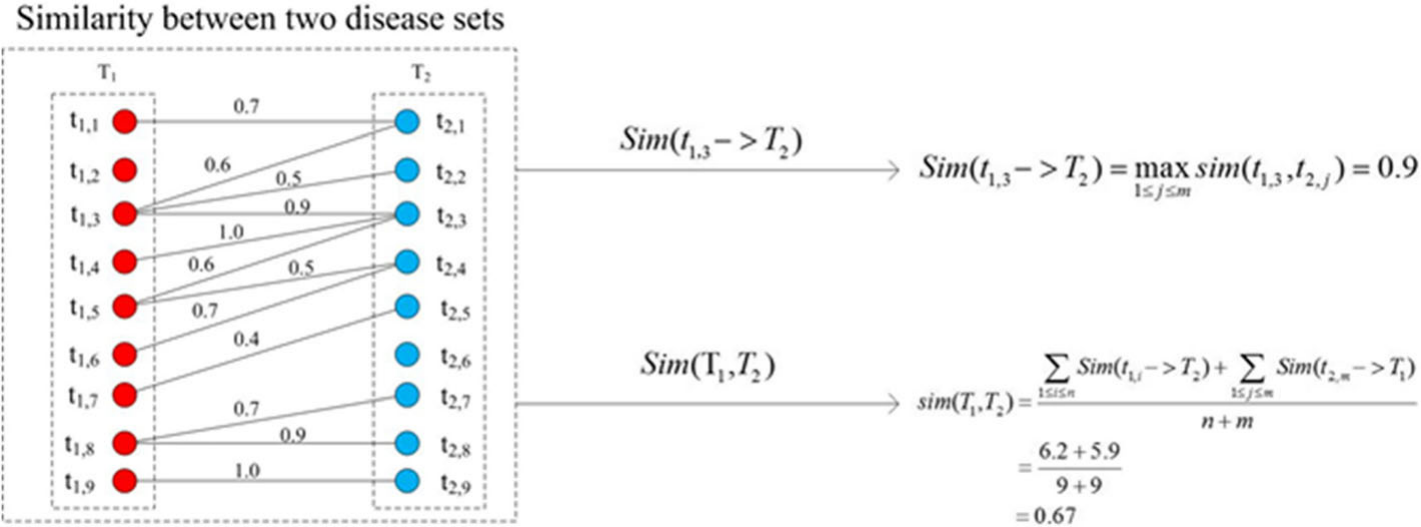
Shows an example of calculating similarity between disease sets T1 and T2

Assuming *T*_*1*_ and *T*_*2*_ are two disease sets, which includes *n*, and *m* diseases, respectively. Similarity between two disease sets (Fig. 2) was defined in the eq. 6 as following:

6 $$ sim\left({T}_1,{T}_2\right)=\frac{\sum \limits_{1\le i\le n} Sim\left({t}_{1,i}->{T}_2\right)+\sum \limits_{1\le j\le m} Sim\left({t}_{2,j}->{T}_1\right)}{n+m}, $$

where *t*_*1,i*_, and *t*_*2,j*_ represent the *i*th and *j*th diseases of *T*_*1*_ and *T*_*2*_, respectively. *Sim*(*t*_1, *i*_ − > *T*_2_) represents similarity from a disease term of *T*_*1*_ to *T*_*2*_. Taken *t*_*1,1*_ for example, the eq. 7 gives the definition as following:

7 $$ Sim\left({t}_{1,1}->{T}_2\right)=\underset{1\le j\le m}{\max } sim\left({t}_{1,1},{t}_{2,j}\right). $$

### Method for predicting disease-related lncRNAs and miRNAs

Disease-related lncRNAs and miRNAs were indicated applying a global network ranking algorithm called random walk with restart (RWR) [40]. The random walker starts from one or several seed nodes and then randomly transits to neighboring nodes considering the probabilities of the edges connected the two nodes. And the probability of returning to the seed node is supposed as *γ*. Then, RWR algorithm can be defined as following:

8 $$ {\mathrm{P}}_{t+1}=\gamma {\mathrm{P}}_0+\left(1-\gamma \right){\mathrm{AP}}_t, $$

where *P*_*0*_ represents the initial probability vector, which changes with the step *t* and the probability *γ*, *P*_*t*_ is a vector in which the *i*th element represents the probability of finding the walker at node *i* and step *t*, A indicates the column-normalized adjacency matrix of the network. The algorithm was implemented until the difference between *P*_*t*_ and *P*_*t + 1*_ falling below 10^−10^, which indicates all the nodes’ status become stable.

Based on our method, researchers can predict novel lncRNA-disease and miRNA-disease associations based on RWR. Firstly, a LSN (MSN) could be constructed for RWR. A lncRNA (miRNA) has associations with a set of diseases. Hence, similarity between two lncRNAs (miRNAs) could be computed based on their related disease sets, which promotes to construct a LSN (MSN). Then, lncRNAs (miRNAs) could be scored for each disease based on RWR, in which the known lncRNAs (miRNAs) of a disease are considered as seed nodes. For each disease, the unknown lncRNAs (miRNAs) of it could be scored. After ranking the lncRNAs (miRNAs) based on the scores, disease-related lncRNAs (miRNAs) are finally predicted.

### Method for validating the performance of *InfDisSim*

**Fig. 3:**
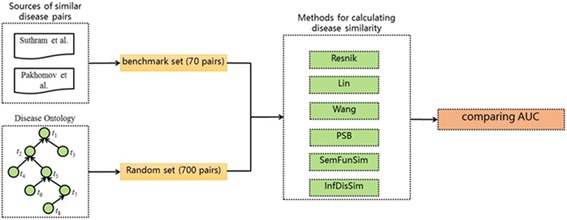
The process of performance evaluation. AUC represents the area under the receiver operating characteristic curve

Figure 3 shows the process of performance validation. At the beginning, a benchmark set including 70 pairs between 47 diseases was derived from two public articles respectively(Additional file 4). One of them is Suthram et al.’s study [41], by which similar pairs of diseases were recognized according to the disease-related mRNA expression data and the human protein interaction network. The other is Pakhomov et al.’s study [42], in which similar pairs of diseases were manually checked by experts in related fields. Then, a random set involving ten times of the benchmark set was obtained from DO. After that, the similarities of benchmark set and random set were calculated by the state-of-art methods including Resnik’s, Lin’s, Wang’s, PSB, SemFunSim, and *InfDisSim*. Finally, the receiver operating characteristic (ROC) curve was drew for assessing the performance of these methods. Furthermore, the experiment was iterated 100 times, and the average of the region under the ROC curve (AUC) for each method was obtained.

## Results

### Performance evaluation based on benchmark set

**Fig. 4:**
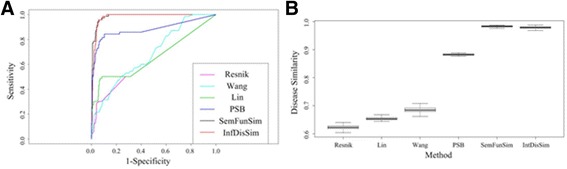
AUC analysis based on the benchmark set. **a** ROC curves of the state-of-art methods. **b** AUCs of 100 iterators

ROC curves of the state-of-art methods based on a benchmark set and a random set are shown in Fig. 4a. The figure indicates that the AUCs of Resnik’s, Lin’s, Wang’s, PSB, SemFunSim and *InfDisSim* are 0.6283, 0.6586, 0.6837, 0.8807, 0.9843, and 0.9786, respectively. Obviously, the performances of three typical methods involving Resnik’s, Lin’s, and Wang’s methods are almost the same. And all of these three methods perform generally. By the contrast, three novel methods that predicted more disease gene associations and gene interactions perform superior, of which the performances of SemFunSim and *InfDisSim* are the best and nearly the same.

Resnik’s, Lin’s, and Wang’s methods concentrated on sematic associations. Few of disease gene associations were employed by these three methods. With more and more disease gene associations and gene interactions identified, it is easier to study similarity between diseases in molecular level. Fortunately, three methods including PSB, SemFunSim, and *InfDisSim* have intergrated these associations into semantic associations. It is easy to find the interactions between genes including mRNA co-expression, protein-protein interaction, protein complex, and so on. Although PSB method only applied co-occurrenced biological process of genes, its performance has already been improved. To enhance the performance, SemFunSim and *InfDisSim* methods employed comprehensive gene functional associations from two different views. And both of these two methods perform excellently.

Figure 4b shows the AUCs of the 100 iterators, which are consistent with the Fig. 4a. From this figure, the average AUCs of the 100 iterators are 0.6223, 0.6538, 0.6851, 0.8824, 0.9832, and 0.9788, respectively.

### Relationship between disease similarity by *InfDisSim* and co-occurrence drugs

**Fig. 5:**
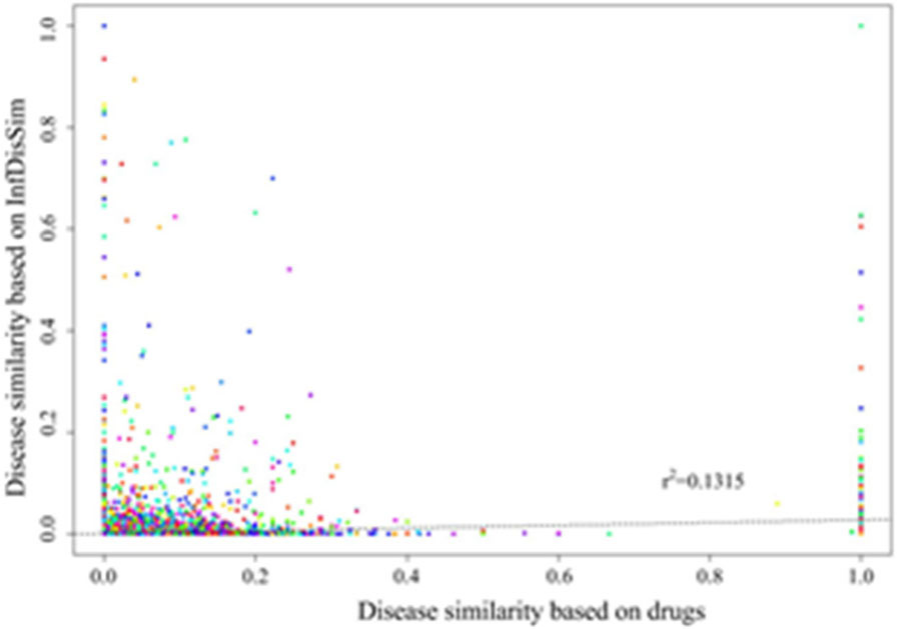
The relationship between disease similarity based on InfDisSim and co-occurrence drugs

Previous studies have indicated that similar diseases could have common therapeutic drugs [9, 10]. Therefore, it is possible that similar diseases tend to have more co-occurrence drugs. To prove this, we discuss the relationship of disease similarity by *InfDisSim* with co-occurrence drugs. In this study, we employed the Jaccard index as the measure for disease similarity by drugs. As a consequence, *InfDisSim* disease similarity showed significant positively correlated with the co-occurrence drugs (Pearson correlation γ^2^ = 0.1315, *p* = 2.2e-16; Fig. 5). Results demonstrate that disease similarity detected by our method is correlated with co-occurrence drugs, which have a very strong correlation with disease similarity.

### Application of disease similarity to the prediction of disease-related lncRNAs

For the sake of showing the usefulness of disease similarity computed by our InfDisSim, we firstly constructed a lncRNA similarity network (LSN) based on disease similarity, and then identified disease-related lncRNAs based on LSN. The similarity of each pair of 111 lncRNAs was computed using the eq. 6. After that, the z-score of each pair of lncRNAs was computed based on these scores. Then, each similarity score gained a one-sided *P*-value. Finally, all of these lncRNA similarity scores were appiled to construct LSN (Additional file 5).

**Fig. 6:**
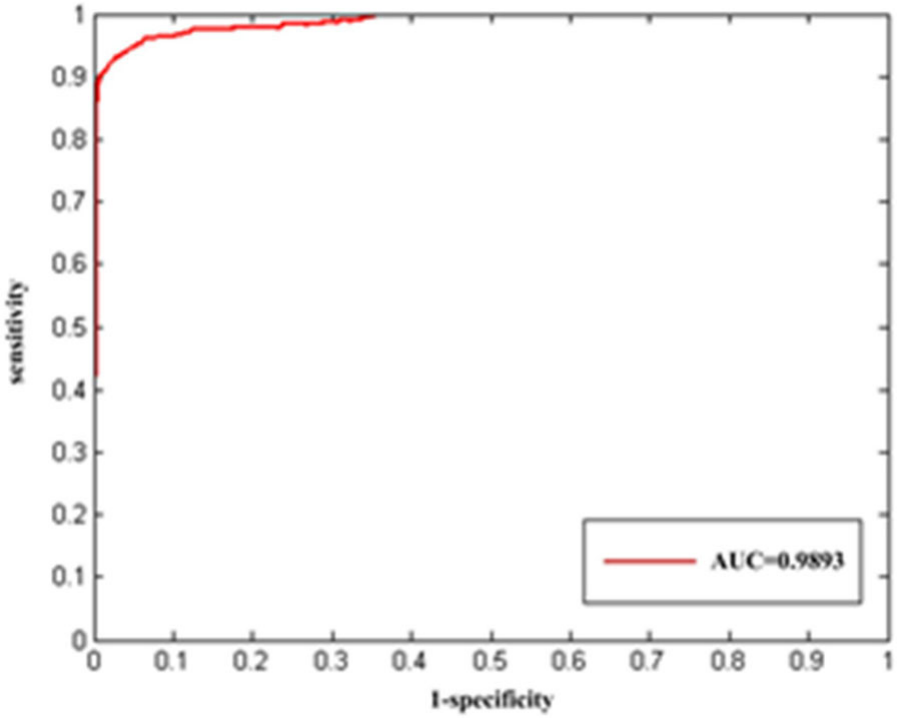
The ROC curve of our method based on leave-one-out cross validation on experimentally verified lncRNA-disease associations

LSN was further employed to predict disease-related lncRNAs employing RWR algorithm. According to the known 331 associations between 125 diseases and 111 lncRNAs, the performance of the LSN was assessed by leave-one-out cross validation. Finally, an AUC of 0.9893 was obtained (Fig. 6).

### Application of disease similarity to the prediction of disease-related miRNAs

We also utilized the disease similarity to construct a MSN and predict disease-related miRNAs based on the network. Here, we calculated similarity of each pair of 265 miRNAs and corresponding one-sided P-value. All of these miRNA similarity scores were employed to construct MSN (Additional file 6) for predicting disease-related miRNAs. The performance of the MSN was assessed by leave-one-out cross validation. As a result, we got an AUC of 0.9007.

## Discussion

To identify the disease-related ncRNAs, including lncRNAs and miRNAs, we presented a novel method based on disease similarity using a random walk. With the high AUC performance of predicting disease-related miRNAs and lncRNAs (0.9893, 0.9007), the proposed methods in this paper may also be applied to predict other disease-related modules, e.g. SNP and risk pathways [43, 44].

## Conclusions

In this study, we presented a novel method, *InfDisSim*, to figure out disease similarity by semantic association and disease-related genes. In time of computing similarity based on genes, information flow was modelled into a comprehensive GFN, which is constructed by integrating multiple interactions involving mRNA co-expression, protein-protein interaction, protein complex, and so on. In the precious study, SemFunSim has introduced the interactions of pair-wise genes between different gene set. Here, the whole network was fully employed based on information flow. It introduced a novel view to compute disease similarity.

The performance of *InfDisSim* was validated employing the benchmark set. The high AUC (0.9786) indicates its excellent performance. Then, we assessed the observation that similar diseases could have common therapeutic drugs. Finally, *InfDisSim* disease similarity was significant positively correlated with the co-occurrence drugs (Pearson correlation γ^2^ = 0.1315, *p* = 2.2e-16; Fig. 5). Therefore, *InfDisSim* disease similarity could be utilized to predict potential associations between diseases and drugs.

lncRNA similarity and miRNA similarity could be computed based on *InfDisSim* disease similarity. Here, for all the pairs of lncRNAs (miRNAs), which was applied to construct a LSN (MSN), we calculated their similarities. The network was further used to predicate disease-related lncRNAs (miRNAs). As a result, the high AUC (0.9893, 0.9007) illustrates that the LSN (MSN) is very appropriate for predicting potential associations between diseases and lncRNAs (miRNAs) based on RWR.

## Additional files

Additional file 1: Disease-gene associations. (TXT 2080 kb)

Additional file 2: Disease-lncRNA associations. (TXT 3 kb)

Additional file 3: Disease-miRNA associations. (TXT 132 kb)

Additional file 4: Benchmark set of similar disease pairs. (TXT 11 kb)

Additional file 5: lncRNA functional similarity scores. (TXT 3310 kb)

Additional file 6: miRNA functional similarity scores. (TXT 9539 kb)
